# A protocol to determine the acceptability and feasibility of a pilot intervention emergency department virtual observation unit fall prevention program

**DOI:** 10.1186/s40814-024-01502-7

**Published:** 2024-05-18

**Authors:** Abigail E. Jones, Maura Kennedy, Emily M. Hayden, Kei Ouchi, Kalpana N. Shankar, Anita Chary, Angel Li, Kara Mc Loughlin, Benjamin White, Esteban Franco-Garcia, Vanessa Dellheim, Shan W. Liu

**Affiliations:** 1 Department of Emergency Medicine, Massachusetts General Hospital, Boston, USA; 2 Department of Emergency Medicine, Massachusetts General Hospital, Harvard Medical School, Boston, MA USA; 3 Department of Emergency Medicine, Brigham and Women’s Hospital, Boston, USA; 4 Departments of Emergency Medicine and Medicine, Section of Health Services Research, Baylor College of Medicine, Houston, TX USA; 5 Valley Health System, Las Vegas, NV USA; 6 Beaumont Hospital, Dublin, Ireland; 7 Department of Internal Medicine, Division of Palliative Care and Geriatric Medicine, Massachusetts General Hospital, Boston, USA; 8 Massachusetts General Hospital, Boston, USA

**Keywords:** Feasibility, Falls, Geriatric, Transitions of care

## Abstract

**Background:**

As a third of all community dwellers aged 65+ fall each year, falls are common reasons for older adults to present to an Emergency Department (ED). Although EDs should assess patients’ multifactorial fall risks to prevent future fall-related injuries, this frequently does not occur. We describe our protocol to determine the feasibility, acceptability, and safety of a pilot ED Virtual Observation Unit (VOU) Falls program.

**Methods:**

To ensure standardized conduct and reporting, the Standard Protocol Items for Intervention Trials (SPIRIT) guidelines will be used. The VOU is a program where patients are sent home from the ED but are part of a virtual observation unit in that they can call on-call ED physicians while they are being treated for conditions such as cellulitis, congestive heart failure, or pneumonia. A paramedic conducts daily visits with the patient and facilitates a telemedicine consult with an ED physician. VOU nursing staff conduct daily assessments of patients via telemedicine. The ED VOU Falls program is one of the VOU pathways and is a multi-component fall prevention program for fall patients who present after an ED visit. The paramedic conducts a home safety evaluation, a Timed Up and Go Test (TUG). During the VOU visit, the ED physician conducts a telemedicine visit, while the paramedic is visiting the home, to review patients' fall-risk-increasing drugs and their TUG test. We will determine feasibility by calculating rates of patient enrollment refusal, and adherence to fall-risk prevention recommendations using information from 3-month follow-up telephone calls, as well as qualitative interviews with the paramedics. We will determine the acceptability of the ED VOU Falls program based on patient and provider surveys using a Likert scale. We will ask VOU nursing staff to report any safety issues encountered while the patient is in the ED VOU Falls program (e.g., tripping hazards). We will use the chi-square test or Fisher’s exact test for categorical variables, Student’s *t*-test for continuous variables, and Mann-Whitney for nonparametric data. We will review interview transcripts and generate codes. Codes will then be extracted and organized into concepts to generate an overall theme following grounded theory methods. This is a pilot study; hence, results cannot be extrapolated. However, a definite trial would be the next step in the future to determine if such a program could be implemented as part of fall prevention interventions.

**Discussion:**

This study will provide insights into the feasibility and acceptability of a novel ED VOU Falls program with the aim of ultimately decreasing falls. In the future, such a program could be implemented as part of fall prevention interventions.

**Supplementary Information:**

The online version contains supplementary material available at 10.1186/s40814-024-01502-7.

## Background

Falls are the second leading cause of unintentional deaths from injury globally [1]. There are approximately 2.2 million ED visits annually for unintentional falls among those aged 65 and above in the USA [2]. Falls among older adults (aged 65 and older) are common, costly, can result in serious injury, and adversely impact the quality of life for older adults [2, 3]. A fall is an event that results in a person coming to rest inadvertently on the ground or floor or other lower level [1, 4]. Approximately 10% of falls are associated with a significant injury [5]. Furthermore, over 650,000 patients are hospitalized for unintentional falls [2]. The number one cause of death due to injury in the older population is unintentional falls, with 10,000 dying annually [6]. Falls lead to health decline in patients and cause social isolation, loss of confidence [7, 8], and an increased risk of admission to nursing homes [9]. Currently, the estimated medical cost of fall-related injuries in the USA is approximately $34–50 billion annually, which will increase as the population ages [10, 11]. Older adults make up 28 million ED visits each year [12], and a fall in an older adult increases the risk of a future fall. In our previous study, 23% of patients had a recurrent fall within 6 months of their initial ED visit [13]. Although older adults frequently present to the ED after a fall, the ED rarely assesses patients’ many intrinsic and extrinsic reasons for a fall, thus missing opportunities to prevent future falls [13, 14].

In a prior study, ED clinicians (physicians and advanced practice providers) have reported they are not willing to spend much time on fall risk identification and management, given tremendous time pressures and ED crowding, and a potential lack of understanding regarding the implications of a fall on older adults [15]. However, interventions during or soon after an ED visit might be a window of opportunity, given patients might be open to intervention at that time [16]. The American and British Geriatric Societies and Geriatric Emergency Department Guidelines recommend a comprehensive fall risk evaluation for patients after a fall [17, 18]. Studies have shown that interventions such as exercise and environmental assessment, and referral to relevant services after interdisciplinary fall risk assessment, reduce injurious falls or future falls [19, 20]. However, other systematic reviews have not found evidence supporting ED-based screening or fall prevention services initiated in the ED for older adults who present to the ED with a fall [16, 21]. Harper et al found that ED fall programs did not reduce the proportion of older adults who had future falls but multifactorial interventions did reduce fall-related injuries [22].

Recently, there has been an increase in home hospital care and telehealth usage [23–32]. Our hospital created a Virtual Observation Unit (VOU) in January 2022 to provide observation-level care for ED patients in their homes. The VOU includes ED nurses communicating with the patient via phone or video, community paramedics for in-home evaluations, administrative staff coordinating care, and emergency medicine physicians conducting and overseeing video visits. Importantly, the paramedics bring a tablet and mobile hotspot into the patients’ home to ensure telehealth video visits are not reliant on the patients’ ability to use a tablet or their access to the internet [33]. To better assess and manage future fall risk among older patients who present to the ED with a fall, we developed a novel pilot ED VOU Falls program within our VOU. This multi-component program aims to complete a fall risk assessment in patient homes and to develop a personalized fall risk-reducing plan for each patient adapted from the Center for Disease Control and Prevention STEADI program [34]. ED VOU Falls program components, in addition to those outlined above which are provided to all VOU patients, include an examination for commonly prescribed fall risk-increasing-drugs such as loop diuretics, opioids, antiepileptics, and benzodiazepines [35, 36], conduct of a home safety evaluation, and conduct of a functional Timed Up and Go Test [TUG [37]]. VOU personnel (community paramedics and emergency medicine physicians) who will work in the ED VOU Falls program will be trained (via an online training module or lecture) in relevant content specific to assessing fall risk (e.g., application of the TUG, fall-risk-increasing-drugs) prior to participating in the ED VOU Falls program. This pilot study will assess the acceptability, feasibility, and safety of the ED VOU Falls program through surveys of patients, caregivers, and providers, and review of electronic health record (EHR) data. We will compare outcomes at 3 months in study patients vs. a comparison group of eligible patients who were not admitted to the ED VOU Falls program.

## Methods

### Study setting

This protocol describes how we aim to evaluate the feasibility, acceptability, and safety of a pilot intervention ED VOU Falls program among 100 patients (50 who receive the intervention and 50 in a comparison group) who visit a Level 1, urban teaching hospital ED in the Northeastern U.S. To ensure standardized conduct and reporting, the Standard Protocol Items for Intervention Trials (SPIRIT) guidelines will be used [see Additional file 1 for checklist) [38]]. The study site’s Institutional Review Board (IRB) reviewed this protocol and deemed it to qualify as an exempt protocol (2023P000065).

### Study population and eligibility

Patients will be enrolled between 8 am and 8 pm when Research Assistants (RAs) are available in the ED. The inclusion criteria will be patients who are

- 65 years and older with a complaint related to a fall
- Able to ambulate by themselves or with their baseline device (typically ED nurses make sure patients are able to ambulate independently before allowing them to go home unless patients are unable to ambulate at their baseline).
- Deemed safe to go home by clinicians
- Reside within the geographic catchment area for the ambulance group that provides the paramedics.

Exclusion criteria will be patients who

- Reside in a healthcare facility (e.g., nursing home, psychiatric facility, acute rehabilitation); patients residing in an assistive living or adult family home would not be excluded
- Have active psychiatric concerns or substance use (e.g., needing potentially acute agitation control, psychiatric evaluation or addictions consult), and have high-risk clinical features
- May need a higher level of care (e.g., needing rehabilitation placement, medical admission)

The comparison group will consist of patients who are not enrolled in the program because of geography or enrollment hours, and who consent to participate in follow-up phone calls.

### Recruitment and enrollment

**Fig. 1:**
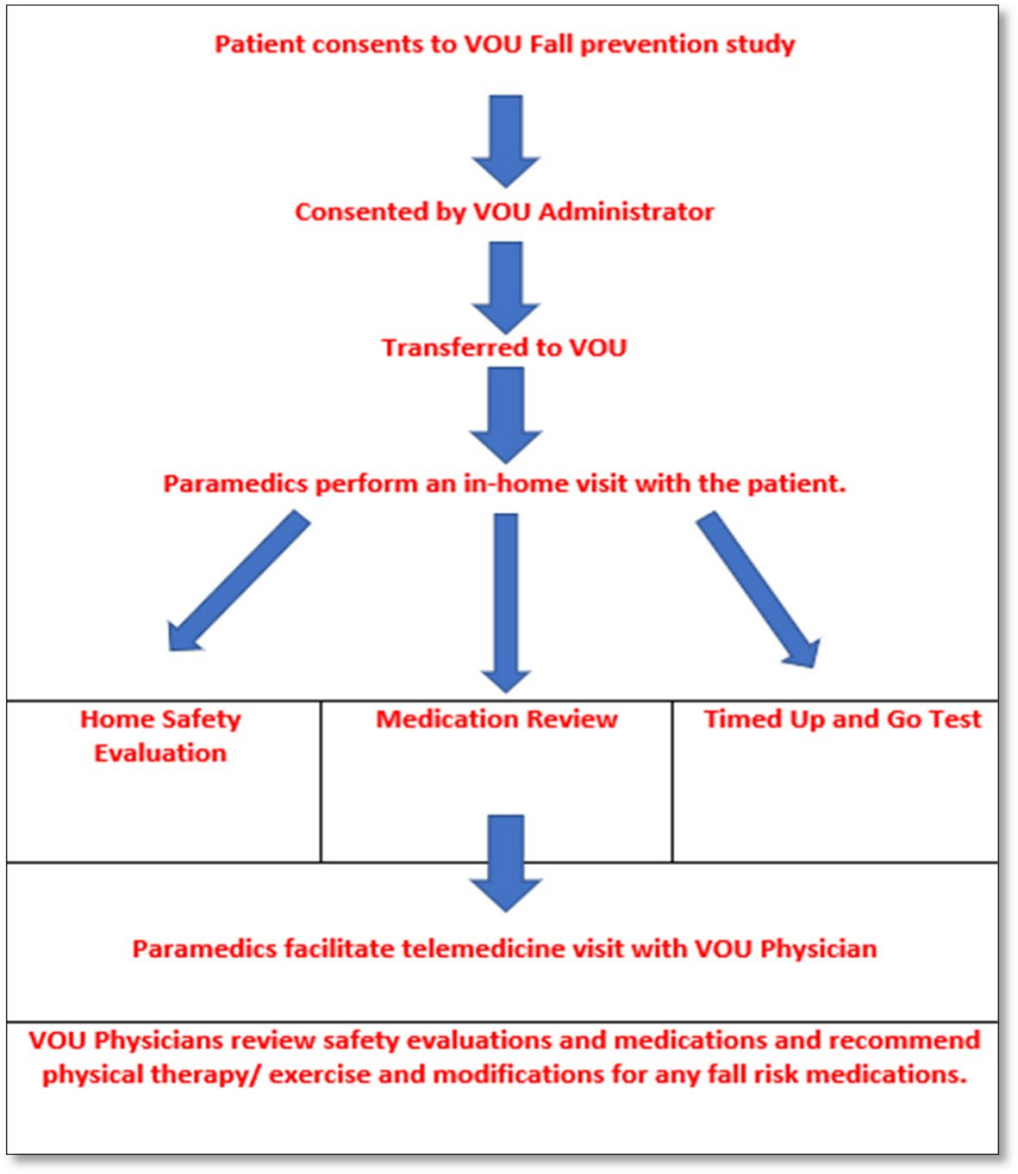
Falls virtual observation unit diagram

Initially, patients will be evaluated and managed in the ED for any injuries and medical etiologies for their falls (see Fig. 1). ED-based RAs will screen the ED census list for patients 65 and older who may have an acute fall-related complaint and will likely be discharged. The RAs will look at triage chief complaints such as slips, trips, or falls following Goldberg et al.’s ED fall protocol [39]. RAs will approach the ED clinicians to ask if an identified patient meets inclusion/exclusion criteria and would be appropriate for the ED VOU Falls program. Clinicians are aware of patient criteria for the pre-existing VOU, and we will educate clinicians about additional criteria that apply to the subset of patients who will be considered for the ED VOU Falls program. Study criteria will be included in the resource repository that all VOU clinicians use during their VOU shifts. ED clinicians who do not work in the VOU will have education via lectures. If the patient is eligible and willing to participate in the study, the Six-item Cognitive Impairment Test (6-CIT) will be administered. We chose the 6-CIT as it is brief and feasible to use in the busy, loud ED. Following Salis et al.’s study on the best 6-CIT cut-off, if a patient has a score greater than or equal to 8 on the 6-CIT, caregiver consent will be required [40]. If a caregiver is not present, we will call the caregiver for consent. The RA will review and provide the IRB fact sheet to the patient. This IRB fact sheet explains the objectives, design, benefits, and risks pertaining to the study (see Additional file 2). Subsequently, the RA will obtain informed verbal consent from the patient or the patient’s caregiver. As patients can participate in the ED VOU Falls program without participating in our feasibility study, the VOU administrator will follow the normal procedure of consenting patients to participate in the VOU. Patients will be given the telemedicine equipment and discharged from the inpatient ED to their homes under the care of the ED VOU Falls program.

### Procedures

A community paramedic will perform an in-home visit with the patient within the next 24 h and will conduct a home safety evaluation and medication system assessment (Additional file 3), and administer a TUG. For the latter, the paramedics will use two cones and a 10-foot string to measure landmarks for the patient. Subsequently, the paramedic will facilitate a telemedicine visit with the emergency medicine physician. The physician will review the results of the paramedic safety evaluation, and TUG test and recommend to the patient any ways to make the home safer and/or refer to physical therapy as needed. Following the American Society of Consulting Pharmacists-National Council on Aging Falls Risk Reduction Toolkit which is a companion to the Center for Disease Control and Prevention (CDC) STEADI Toolkit, the physician will review the patient’s medications and identify any fall risk-increasing-drugs such as anticholinergic (eg diphenhydramine), dopaminergic, opioids, antiepileptics, and benzodiazepines [36, 41]. Specifically, the physician will recommend physical therapy for patients with a TUG test time greater than or equal to 12 s, following the CDC STEADI algorithm [34]. The physician will also communicate with the patient’s primary care provider (PCP) if there are any fall-risk-increasing drugs identified. If a PCP is not in our healthcare system, the physician will instruct the patient to communicate with his/her PCP to discuss the fall risk-increasing drug. The paramedic and physician will encourage all patients to exercise. We will follow the ASPIRE Exercise recommendations for fall prevention that include standing or chair exercises based on the Otago Exercise Program [42, 43]. The ED VOU Falls program team will then determine if the patient can be discharged from their services. Typically, we will expect only one visit from the paramedic. Patients can decline to participate in the ED VOU Falls program intervention at any time.

An RA will administer the patient or caregiver an initial follow-up questionnaire (see below for more details) over the phone 1–2 days after the patients’ ED VOU Falls program discharge. RAs will call up to three times to get responses and collect reasons for refusals. Three months after the patients’ ED VOU Falls program participation, the RAs will telephone the patient to ask if they changed medications, exercised, changed any safety risks in their homes, or followed up with physical therapy (see Additional file 4). The RAs will also ask about any return visits to the ED (at the study site or elsewhere), and any other reported safety issues during the 3-month period. To increase adherence, those who complete 3-month follow-up will receive $40 either in the form of a gift certificate or check. Participation in the ED VOU Falls program will not preclude concomitant care or interventions. The follow-up questions will be adapted from previous ED fall studies [44, 45].

### Data collection

RAs will collect patient data, including age, gender, race, insurance, primary care doctor, education, primary spoken language, and marital status from the study site’s electronic health record system (Epic, Madison, WI). The RA will also collect Charlson comorbidities [46, 47], and determine the ED return rate for falls and non-fall complaints within 3 months post-ED VOU Falls program discharge in Epic.

#### Surveys

The RA will administer the initial ED VOU Fall follow-up and VOU attending physician questionnaire with a mix of questions using a 1–5 Likert scale and open-ended responses [48, 49]. Based on questions adapted from the theoretical framework of acceptability (TFA) [50], the questionnaires (see Additional files 4 and 5) will examine how the patients and VOU physicians rate the comfort, effort, fairness, acceptability, feasibility, and safety of the program. While the VOU physicians are part of the intervention, their perceptions of the program are critical for the continued implementation and success of such a program. It is important to capture many stakeholders’ acceptability of the program.

As a part of the evaluation of the safety of implementing a fall management program in the existing VOU service, the PI will ask VOU nurses every 2 weeks if there are any safety incidents (e.g., tripping hazards) that have occurred while any patient is in the ED VOU Falls program. Any safety issues will lead to the review of the safety of the VOU intervention. We will assess the feasibility of the study in part on patient recruitment rates. When the RA identifies a patient that is eligible for the ED VOU Falls program, the RA will approach the clinician and see if the clinician is willing to admit the patient to the ED VOU Falls program. The number of patients who enroll in the program divided by the number of patients who are approved for the program by the clinician will be calculated as the recruitment rate. We will also record why the clinician did not think a patient was appropriate for the program or the reason for any patient refusals of admittance to the VOU.

Furthermore, we will conduct qualitative interviews of paramedics to determine how comfortable they are with conducting the TUG, home safety evaluation, and how they would improve the program based on questions adapted from the TFA and another paramedic qualitative falls study [50, 51].

Study data will be collected and managed using REDCap (Research Electronic Data Capture), a secure, web-based software platform designed to support data capture for research studies [52, 53]. Data will be stored in REDCAP or on hospital password-protected computers. Only study staff will have study identifiers that link to patient identifiers.

### Outcomes

Ultimately, the ED VOU Falls program aims to identify and ameliorate risks for future falls among patients who have had a fall-related ED visit. However, in a relatively small pilot test, sample sizes are likely too small to detect between-group differences in fall rates. Instead, we will collect data about any subsequent falls but also plan to assess three intermediate outcomes; feasibility, acceptability, and safety. This study will also allow us to calculate baseline risk-mitigating rates between intervention and control groups that will inform sample size calculations for future studies.

We will determine how feasible it is to recruit 50 patients into the ED Falls VOU program over 6 months with our current inclusion/exclusion criteria. We may find that we may need to change our inclusion criteria if there are not enough patients in the geographical catchment area, or if not enough patients who present to the ED after a fall are willing to be part of a virtual observation unit. Furthermore, we will also determine the feasibility of the program based on whether patients report having done anything to change their risk of falling at their 3-month follow-up calls. Feasibility will also include assessing the self-reported ability of paramedics to conduct TUG testing, home safety evaluation, and medication system assessments, as well as VOU attending physician self-report of program feasibility that will be gathered from the previously mentioned interviews and surveys.

We will define acceptability as an average of ≥ 4 on our 1–5 Likert scale survey questions of VOU attendings and patients (range Completely Unacceptable to Completely Acceptable).

Safety will be assessed in several ways. As described above, we will elicit data about return fall-related ED visits from the EHR, and about subsequent falls or other accidents with or without injury from patients or caregivers and from ED VOU Falls program nurses and paramedics. We will also define safety as an average of ≥ 4 on our 1–5 Likert scale survey questions of attending (range Completely Unsafe to Very Safe).

The PI will review each ED VOU Fall study patient’s chart to determine if the home safety and medication safety evaluations were conducted as well as whether the TUG was conducted and PT referral made if appropriate and exercise recommended.

### Data analysis

We will compare the rate of patient-reported fall risk modification between the intervention and control groups at 3 months. We will include a sample size similar to other feasibility studies [54]. If we assume that 0.5 of the intervention group changes their fall risk behavior compared to 0.3 of the comparison group, we will need a sample size of 96 patients to have 80% power to detect the difference with a 95% confidence interval.

We will calculate the rates of patient enrollment refusal. We will calculate differences in demographics between the ED VOU Fall program patients and those who were not enrolled as well as their 3-month fall rates using the chi-square test or Fisher’s exact test for categorical variables and Student’s *t* test for continuous variables. We will impute any missing data.

To measure adherence to fall-risk mitigation recommendations, we will compare rates of patient-reported follow-through of recommendations during 3-month calls. Specifically, for each intervention patient, we will record any recommendations in EHR for (1) home safety, (2) PT, (3) exercise, and (4) medication changes as dichotomous yes/no variables. For survey data, we will calculate median scores and variance using the Mann-Whitney test. We will calculate the frequency of safety events such as tripping and falling that occurred during ED VOU Falls admission).

Paramedic qualitative interviews will be transcribed and codes will be generated inductively by reading and rereading our primary data and deductively from experience and theory [55]. Two study investigators will meet and review transcripts and discuss common concepts and categories. Codes will then be extracted and organized into concepts to generate an overall theme following grounded theory methods [55]. We will conclude the analysis when all interviews are coded and themes analyzed.

## Discussion

We describe the feasibility, acceptability, and safety protocol of a pilot ED VOU Falls program at a tertiary academic hospital in the Northeastern U.S. This study is highly innovative due to the promotion of collaboration across five disciplines and groups, including emergency medicine physicians, paramedics, nurses, patients, and caregivers. It also integrates a distinctive method for providing care remotely using physicians and in-person evaluation with paramedics. We hypothesize that the ED VOU Falls program will improve the frequency with which ED patients undergo fall risk evaluation.

There are some limitations to this study. One of the main limitations may be an insufficient number of patients willing to participate in the ED VOU Falls program. Another limitation is patients and caregivers may provide inaccurate feedback or self-reported changes due to social desirability bias. However, their feedback is crucial despite this potential bias. Additionally, paramedics may initially feel uncomfortable evaluating fall patients. However, it is believed that a simple TUG test can be taught to paramedics as prior studies have demonstrated that non-medical personnel, such as research assistants, can perform this test [37, 56]. Also, paramedics may not be experienced in doing home safety evaluations. However, when designing this program, our paramedic group provided a home safety protocol that they had previously used. Furthermore, paramedics will receive training on how to use the home safety checklist and how to conduct the TUG test. While ideally we would be able to conduct observations of each paramedic doing an ED VOU Fall evaluation in the patient’s home, there are insufficient funds to do so at this time. However, as mentioned, each study subject’s chart will be reviewed to determine whether paramedics and VOU physicians implemented all elements of the protocol. Another potential limitation of this study is a loss of follow-up. For instance, ED patients who present with a fall have up to 15% 1-year mortality rate [44, 57]. Since these patients are discharged from the ED and can ambulate safely, we anticipate the mortality rate will be lower, and the loss to follow-up rate will be minimal. In a previous study, our follow-up rate was 86.3% using phone calls and medical records to determine if the patient returned to the ED, was hospitalized, or reported a fall [58]. If the loss to follow-up rate is higher than anticipated when conducting the 3-month follow-up calls, we will extend enrollment to more patients. Also, there may be survey bias from VOU providers in that only possibly those with strong feelings may be motivated to fill out the survey, thus biasing results. Also, we acknowledge there may be selection bias in referring patients to the ED VOU Falls program if physicians work some shifts in the ED but are also part of the intervention as ED VOU Fall telemedicine providers. Furthermore, given our program is part of a tertiary, urban teaching hospital, it may be not generalizable to other sites.

Falls are common, costly, and dangerous. This study will provide insights into the feasibility, acceptability, and safety of a novel ED VOU Falls program that can inform the subsequent design of a larger fall intervention that could be assessed for its ability to decrease recurrent fall-related injuries.

### Supplementary Information

Additional file 1: SPIRIT 2013 Checklist: Recommended items to address in a clinical trial protocol and related documents*

Additional file 2: IRB fact sheet

Additional file 3: Paramedic Interview Questions

Additional file 4: VOU Fall form

Additional file 5: Patient follow up script

Additional file 6.

## Data Availability

Not applicable.

## Abbreviations

ED: Emergency Department
VOU: Virtual Observation Unit
RA: Research Assistant
TUG Test: Timed Up and Go Test
TFA: Theoretical Framework of Acceptability
6-CIT: Six-Item Cognitive Impairment Test
