# Supplementary material for: A protocol to determine the acceptability and feasibility of a pilot intervention emergency department virtual observation unit fall prevention program
Source: Pilot Feasibility Stud. 2024 May 18;10:79. doi: 10.1186/s40814-024-01502-7 (PMC11102199; doi:10.1186/s40814-024-01502-7)
Supplement: Supplementary file 2 — Additional file 2: IRB fact sheet [file 40814_2024_1502_MOESM2_ESM.docx]

**Determining the Acceptability and Feasibility of an Emergency Department Virtual Observation Unit Fall Prevention Program**

**PATIENT/Caregiver Information form**

Thank you for agreeing to learn more about our research study.

We are conducting this study to investigate new ways of decreasing fall risk. Our Emergency Department has created the Virtual Observation Unit Prevention Program and our study aims to determine whether this program is acceptable and feasible.

In this study, the research assistant will ask you if you are willing to be admitted to the Virtual observation unit (VOU) fall program. Our study seeks to understand how to improve this program and to see if it leads to a decrease in falls at 3 months. If you are admitted to the VOU, a research assistant will call you 1-2 days after your virtual observation unit visit to ask your views about the program. Participants who are admitted to the VOU and those who are NOT admitted to the VOU will be called by our research assistant in 3 months to see if you have fallen again and if you have been able to change your fall risk. These calls will take less than 10 minutes.

We will also review your medical record to obtain basic demographic information, your medical conditions, your ED diagnosis and whether you fell again after your ED visit. None of your personal health information will be shared with anyone outside of the study. Results will be kept confidential.

Decreasing fall risk will decrease future falls and that by participating in this program, you can help us improve the program. There is no direct benefit to you to being interviewed, but there could be benefit to other patients in the future. Your treatment today will not be delayed or affected by this research study. Participation in the study is voluntary and will in no way affect anything else involved in your care. If you decide not to participate, it will not affect your care.

We hope to enroll 350 subjects. We will mail a $40 gift certificate either to you (the patient) OR the caregiver if you complete the study, including the 3-month follow up call.

If you have any questions regarding the study contact:

Dr. Shan Liu, Department of Emergency Medicine, Massachusetts General Hospital, Five Emerson Place, 119c, Boston MA 02114, 617.726.4809. Dr. Liu can also be reached through the MGH Page Operator at 617.726.2000.

If you’d like to speak to someone not involved in this research about your rights as a research subject, or any concerns or complaints you may have about the research, contact the Mass General Brigham IRB at (857) 282-1900.

We are required by the Health Insurance Portability and Accountability Act (HIPAA) to protect the privacy of health information obtained for research. This is an abbreviated notice, and does not describe all details of this requirement. During this study, identifiable information about you or your health will be collected and shared with the researchers conducting the research. In general, under federal law, identifiable health information is private. However, there are exceptions to this rule. In some cases, others may see your identifiable health information for purposes of research oversight, quality control, public health and safety, or law enforcement. We share your health information only when we must, and we ask anyone who receives it from us to protect your privacy.
