# Supplementary material for: A protocol to determine the acceptability and feasibility of a pilot intervention emergency department virtual observation unit fall prevention program
Source: Pilot Feasibility Stud. 2024 May 18;10:79. doi: 10.1186/s40814-024-01502-7 (PMC11102199; doi:10.1186/s40814-024-01502-7)
Supplement: Supplementary file 3 — Additional file 3: Paramedic Interview Questions [file 40814_2024_1502_MOESM3_ESM.docx]

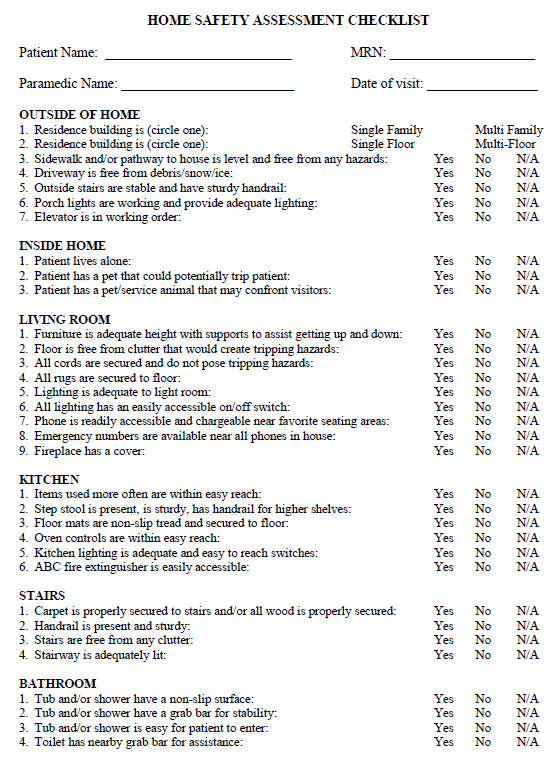


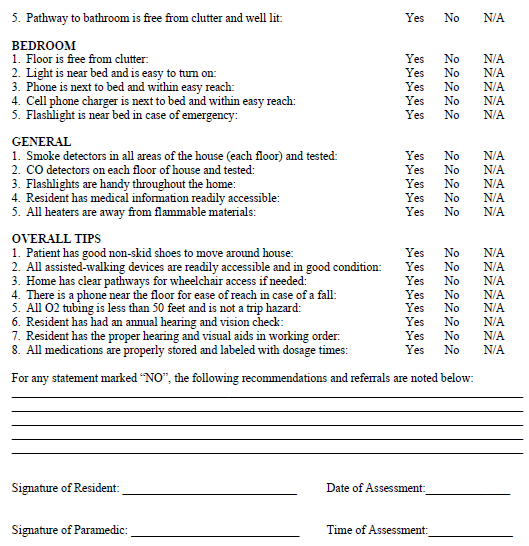


TUG TEST > 12 Sec ? YES NO

Medication System :

1. Does patient have a pill box? Yes No NA
2. Do patient have expired

medications mixed with

unexpired ones? Yes No NA

1. Does patient have an

organized medication Yes No NA

system?

1. Do you have concerns about Yes No NA

patient’s medication system?
