# Supplementary material for: A protocol to determine the acceptability and feasibility of a pilot intervention emergency department virtual observation unit fall prevention program
Source: Pilot Feasibility Stud. 2024 May 18;10:79. doi: 10.1186/s40814-024-01502-7 (PMC11102199; doi:10.1186/s40814-024-01502-7)
Supplement: Supplementary file 4 — Additional file 4: VOU Fall form [file 40814_2024_1502_MOESM4_ESM.docx]

Post ED VOU FALL PATIENT OR CAREGIVER interview (for those who enrolled in the VOU)

“Hello, my name is _________. You (or your family member) were admitted to the ED virtual observation unit recently We are calling to follow up with you. Would you be willing to participate in this follow up call?”

Willing to participate? Yes No

1. Please tell me about your virtual observation unit visit.
2. How comfortable did you feel about the virtual observation unit fall program?

Very uncomfortable Uncomfortable No opinion Comfortable Very Comfortable

1. How much effort did the fall program take?

No effort at all A little effort No opinion A lot of effort Huge effort

1. How fair is the program for fall patients?

Very unfair Unfair No opinion Fair Very fair

1. The virtual observation unit fall program has improved my fall risk

Strongly disagree Disagree No opinion Agree Strongly agree

1. It is clear to me how the virtual observation fall program will help my fall risk.

Strongly disagree Disagree No opinion Agree Strongly agree

1. How confident do you feel about being able to reduce your fall risk?

Very unconfident Unconfident No opinion Confident Very confident

1. Reducing my fall risk interferes with my other priorities

Strongly disagree Disagree No opinion Agree Strongly agree

1. How acceptable was the virtual observation unit fall program to you?

Completely unacceptable Unacceptable No opinion Acceptable Completely acceptable

1. What preferences do you have regarding the recommendations made during the ED VOU Falls program?

Completely unacceptable Unacceptable No opinion Acceptable Completely acceptable

3- Month Follow up Patient Calls

“Hello, my name is _________. You came to the ED, __/__/__. We are calling to follow up with you. Would you be willing to participate in this follow up call?”

Willing to participate? Yes No

1. Have you had any falls since last ED visit? Yes No

How many?________

1. Have you done anything to change your risk of falling? Yes No

If so, what have you done?___

Physical Therapy Yes No

Followed Exercise Recommendation Yes No

Changed medications Yes No

Changed anything to make home safer Yes No

1. Have you returned to the ED since last visit? Yes No

How many times?________

Reason for visit?_________

1. Have you been admitted to the hospital since last visit? Yes No

How many times?_________

Reason for visit?__________

1. How has your health been since your fall? Better Same Worse

Explain________________________-

1. Have there been any safety issues related to the VOU program? (For those patients who enrolled in the VOU)
2. Do you have any questions?

Thank you very much for participating in this study.”
