# Supplementary material for: A protocol to determine the acceptability and feasibility of a pilot intervention emergency department virtual observation unit fall prevention program
Source: Pilot Feasibility Stud. 2024 May 18;10:79. doi: 10.1186/s40814-024-01502-7 (PMC11102199; doi:10.1186/s40814-024-01502-7)
Supplement: Supplementary file 5 — Additional file 5: Patient follow up script [file 40814_2024_1502_MOESM5_ESM.docx]

**Emergency Department Falls Survey for VOU Physicians**

***We (Shan Liu, MD, Maura Kennedy, MD, Emily Hayden MD) are conducting a 5-***

***minute research survey to identify issues related to the ED VOU Falls Program.***

***The survey is anonymous and your participation is voluntary. Three survey participants will be randomly selected to receive one of five $40 checks. Otherwise, there is no payment for answering these questions. The responses from this survey will be analyzed with the intention to publish the results of this survey in a peer-reviewed manuscript.***

Have you taken care of a patient in the Virtual Observation Unit?

Yes No

1. How comfortable do you feel about managing patients in the ED VOU Falls program?

Very uncomfortable Uncomfortable No opinion Comfortable Very Comfortable

1. How much effort did the ED VOU Falls program take?

No effort at all A little effort No opinion A lot of effort Huge effort

1. How fair is the program for fall patients?

Very unfair Unfair No opinion Fair Very fair

1. The ED VOU Falls program improves patient fall risk

Strongly disagree Disagree No opinion Agree Strongly agree

1. It is clear to me how the ED VOU Falls program will help patient fall risk.

Strongly disagree Disagree No opinion Agree Strongly agree

1. How confident do you feel about being able to reduce a patient’s fall risk?

Very unconfident Unconfident No opinion Confident Very confident

1. Reducing patient’s fall risk interferes with other ED priorities

Strongly disagree Disagree No opinion Agree Strongly agree

1. How acceptable was the ED VOU Falls program to you?

Completely unacceptable Unacceptable No opinion Acceptable Completely acceptable

1. How feasible is the ED VOU Falls program?

Completely not feasible No feasible No opinion Feasible Very feasible

1. How safe is the ED VOU Falls program?

Completely unsafe Unsafe No opinion Safe Very safe

1. How would you improve the ED VOU Falls program?
2. What the best aspects of the ED VOU Falls program?
3. What are the challenges/barriers of the ED VOU Falls program?

Age: ___________

Ethnicity

1 = White

2 = Asian

3 = American Indian/Alaskan Native

4 = Native Hawaiian or Pacific Islander

5 = African American or Black

6 = Other

7 = Unknown
