# Supplementary material for: A protocol to determine the acceptability and feasibility of a pilot intervention emergency department virtual observation unit fall prevention program
Source: Pilot Feasibility Stud. 2024 May 18;10:79. doi: 10.1186/s40814-024-01502-7 (PMC11102199; doi:10.1186/s40814-024-01502-7)
Supplement: Supplementary file 6 — Additional file 6. [file 40814_2024_1502_MOESM6_ESM.docx]

| 1. How has your experience been with geriatric fall patients in general in your career? 2. What role do you think EMS has in assessing fall risk among geriatric patients? 3. What role do you think EMS has in fall prevention among geriatric patients? 4. How many fall patients did you have in the ED Falls VOU Program? 5. How comfortable are you with performing the Timed Up and Go (TUG) test? Explain. 6. How familiar were you with the TUG test before the ED Falls VOU Program ? Explain. 7. How was your experience conducting the TUG test with the ED Falls VOU Program patients? 8. How much effort did it take to perform the TUG test? Explain. 9. Moving to the home safety evaluation, how comfortable are/were you with conducting the home safety evaluation? 10. How long did it take to conduct the home safety evaluation? 11. What were some of the common home safety issues you noted? 12. If you did not have a fall patient, how comfortable would you be in conducting the home safety evaluation? 13. Let’s chat about the medication safety portion of the ED Falls VOU Program. How did you identify whether the medication system was safe? 14. How comfortable were you with identifying whether the patient had a safe medication system? 15. What issues did you experience when evaluating the patient’s medications? 16. How would you improve the ED Falls VOU Program? 17. What the best aspects of the ED Falls VOU Program? 18. What are the challenges/barriers to implementing the ED Falls VOU Program? 19. Did you have any particularly positive experiences while taking part in the ED Falls VOU Program? If so, please describe them: 20. Did you have any particularly negative experiences while taking part in the ED Falls VOU Program? If so, please describe them: 21. How do patients feel about the ED Falls VOU Program? 22. How do you feel about EMS conducting fall prevention work? |
| --- |
